# Safety and pharmacokinetics of motesanib in combination with gemcitabine for the treatment of patients with solid tumours

**DOI:** 10.1038/sj.bjc.6604723

**Published:** 2008-10-28

**Authors:** T J Price, L Lipton, J McGreivy, S McCoy, Y-N Sun, M A Rosenthal

**Affiliations:** 1 The Queen Elizabeth Hospital, 28 Woodville Road, Woodville West, SA 5011, Australia; 2 Department of Medical Oncology, Western Hospital, Gordon St., Footscray, Melbourne, VIC 3011, Australia; 3 Amgen Inc., One Amgen Center Drive, Thousand Oaks, CA 91320, USA; 4 Amgen Inc., South San Francisco, CA 94080, USA; 5 Royal Melbourne Hospital, Grattan Street, Parkville, Melbourne, VIC 3050, Australia

**Keywords:** advanced solid tumours, angiogenesis, gemcitabine, lymphoma, motesanib diphosphate, pharmacokinetics

## Abstract

The aim of this open-label phase 1b study was to assess the safety and pharmacokinetics of motesanib in combination with gemcitabine in patients with advanced solid tumours. Eligible patients with histologically or cytologically documented solid tumours or lymphoma were enroled in three sequential, dose-escalating cohorts to receive motesanib 50 mg once daily (QD), 75 mg two times daily (BID), or 125 mg QD in combination with gemcitabine (1000 mg m^−2^). The primary end point was the incidence of dose-limiting toxicities (DLTs). Twenty-six patients were enroled and received motesanib and gemcitabine. No DLTs occurred. The 75 mg BID cohort was discontinued early; therefore, 125 mg QD was the maximum target dose. Sixteen patients (62%) experienced motesanib-related adverse events, most commonly lethargy (*n*=6), diarrhoea (*n*=4), fatigue (*n*=3), headache (*n*=3), and nausea (*n*=3). The pharmacokinetics of motesanib and of gemcitabine were not markedly affected after combination therapy. The objective response rate was 4% (1 of 26), and 27% (7 of 26) of patients achieved stable disease. In conclusion, treatment with motesanib plus gemcitabine was well tolerated, with adverse event and pharmacokinetic profiles similar to that observed in monotherapy studies.

Angiogenesis, the process by which new blood vessels develop, plays a critical role in tumour development and metastasis by providing tumours with nutrients and oxygen (Folkman and Shing, 1992). The proangiogenic cytokine vascular endothelial growth factor (VEGF) promotes neovascularisation by binding and activating VEGF receptor 2 (VEGFR2) and VEGFR1 (Ferrara *et al*, 2003). Solid tumours also express platelet-derived growth factor receptor (PDGFR) and stem cell factor receptor (Kit); both have been shown to play a role in the pathogenesis of several tumour types (Heinrich *et al*, 2002; Yu *et al*, 2003). Inhibition of angiogenesis has emerged as an effective treatment strategy in some solid tumours (Yang *et al*, 2003; Miller *et al*, 2005; Sandler *et al*, 2006).

Motesanib is a highly selective, oral inhibitor of angiogenesis with direct antitumour activity. It inhibits VEGFR1, 2, and 3 (50% inhibitory concentration (IC_50_)=2, 3, and 6 nM, respectively); and PDGFR (IC_50_=84 nM) and Kit (IC_50_=8 nM), which may confer direct antitumour activity (Polverino *et al*, 2006). In a phase 1 study, motesanib monotherapy in patients with advanced solid tumours had tolerable toxicity and showed encouraging activity against a number of tumour types, including sarcoma, renal cell carcinoma, and thyroid cancer (Rosen *et al*, 2007).

Recent studies have suggested that combination treatment with a VEGF inhibitor plus cytotoxic chemotherapy may result in enhanced efficacy compared with single-agent approaches, whereas maintaining a favourable adverse event profile (Miller *et al*, 2005, 2007; Giantonio *et al*, 2007; Herbst *et al*, 2007; Heymach *et al*, 2007). Gemcitabine, which has activity in many solid tumours, has been combined with the monoclonal anti-VEGF antibody bevacizumab for the treatment of hepatocellular carcinoma or advanced pancreatic cancer (Kindler *et al*, 2005; Zhu *et al*, 2006). The aim of this study was to determine the maximum target dose and the pharmacokinetic profile of motesanib when administered in combination with gemcitabine in patients with advanced solid tumours, and to assess the safety and efficacy of this treatment regimen.

## Materials and methods

### Patients

Eligible adult (⩾18 years) patients had histologically or cytologically documented solid tumours or lymphoma; Eastern Cooperative Oncology Group score zero to two; systolic/diastolic blood pressure ⩽145/⩽85 mmHg; adequate cardiac, hepatic, and renal function; and were candidates for gemcitabine treatment as deemed by the investigator. Patients were ineligible if they had received prior treatment with VEGF inhibitors or gemcitabine, had received systemic chemotherapy within 21 days of study day 1 or strong inhibitors of cytochrome P450 (CYP) 3A4 within 7 days of study day 1. Other main exclusion criteria were: non-small cell lung cancer (NSCLC) with squamous cell histology; large central tumour lesions in the thorax (⩾3 cm and located adjacent to or within the hilum or mediastinum); evidence of active bleeding or bleeding diathesis; and untreated or symptomatic brain metastases. All patients provided written informed consent.

### Study design

This was an open-label, phase 1b, dose-finding study conducted at three centres in Australia. All study procedures were approved by the respective independent ethics committee and/or institutional review board. The primary end point was the incidence of dose-limiting toxicities (DLTs). The secondary end point was assessment of the pharmacokinetic profile of motesanib and gemcitabine, respectively, when administered alone or in combination. The safety end points included incidence of adverse events, serious adverse events, and laboratory abnormalities not defined as DLTs. Exploratory end points included the response rate as defined by modified Response Evaluation Criteria in Solid Tumours (RECIST) (Therasse *et al*, 2000).

### Dose escalation and target dose

Motesanib diphosphate (Amgen Inc., Thousand Oaks, CA, USA) was self-administered continuously by mouth beginning on day 2 of cycle 1. Based on the range of tolerable doses explored in a previous study (Rosen *et al*, 2007), patients were enroled in three sequential, dose-escalating cohorts (up to six evaluable patients each): motesanib 50 mg once daily (QD) (starting dose), 75 mg two times daily (BID), or 125 mg QD. Enrolment in the higher dose cohorts began only if the initial 50-mg QD dose was well tolerated (four or more patients with no DLTs). Dose escalation within cohorts was not permitted. In the 125-mg QD cohort, if two or fewer patients experienced a DLT in the first 4 weeks of treatment this dose would be considered the target QD dose. Gemcitabine (1000 mg m^−2^) was administered intravenously once weekly for 7 weeks followed by 1 week of rest (cycle 1), then once weekly for 3 weeks followed by 1 week of rest (cycles 2–11; all 4-week cycles). Combination treatment continued for up to 48 weeks or until disease progression or unacceptable toxicity occurred.

Dose delays and dose reductions were defined in the protocol for both motesanib and gemcitabine according to treatment-related toxicities.

### Dose-limiting toxicities

DLTs were defined as grade 3 or higher toxicities occurring during cycle 1 that were related to motesanib or to motesanib plus gemcitabine. Fatigue, nausea, diarrhoea, vomiting, neutropenia, febrile neutropenia, thrombocytopenia, anaemia, lymphopenia, alopecia, hypertension, and increased aspartate aminotransferase (AST) or alanine aminotransferase (ALT) were excluded unless the following criteria were met: grade 3 fatigue persisting for ⩾7 days or grade 4 fatigue; grade 3 or 4 nausea, vomiting or diarrhoea despite maximum supportive care; grade 3 neutropenia with fever >38.5°C or grade 4 neutropenia; grade 4 thrombocytopenia persisting for >7 days; grade 4 anaemia; grade 4 hypertension; AST or ALT >10 times the upper limit of normal.

### Safety

Adverse events were collected throughout the study and classified according to the Medical Dictionary for Regulatory Affairs (2008). Severity was graded according to the National Cancer Institute Common Terminology Criteria for Adverse Events, version 3.0 (2006). Blood pressure and haematology were assessed on each day of a gemcitabine infusion. Blood biochemistry was assessed on day 1 of each cycle. Samples for urinalysis were collected during cycles 1, 2, and 3 and every other cycle thereafter.

### Pharmacokinetics

Plasma samples for intensive pharmacokinetic analysis of motesanib were collected on week 1 (day 2) and week 2 (day 1) of cycle 1 at predose and 0.25, 0.5, 1, 2, 4, 6, 8 (a subset of patients), 12 (75-mg BID cohort only), and 24 h postdose. Trough plasma pharmacokinetic samples were collected on weeks 9, 13, 21, 29, 37, and 45 or at the end of treatment. Plasma samples for gemcitabine intensive pharmacokinetic analysis were collected for all dose cohorts on week 1 (day 1) and week 2 (day 1) of cycle 1 at predose and 15, 30, 45 (week 1 only), 60, and 120 minutes after infusion start.

Samples were analysed for motesanib (Amgen Inc., Thousand Oaks, CA, USA) and gemcitabine concentrations (Quest Pharmaceutical Services, Newark, DE, USA) using validated liquid chromatography/tandem mass spectrometry methods with a lower limit of quantitation of 0.5 and 10 ng ml^−1^, respectively.

Noncompartmental pharmacokinetic analyses were performed using WinNonlin Professional software on Citrix (Version 5.1.1, Pharsight Corporation, Mountain View, CA, USA) to estimate the following pharmacokinetic parameters: maximum observed plasma concentration (*C*_max_), time to *C*_max_ (*t*_max_), the terminal-phase elimination half-life (*t*_1/2,z_), observed plasma concentration at 24 h postdose (C_24_) for motesanib, observed plasma concentration at 2 h postdose (C_2_) for gemcitabine, and area under the concentration *vs* time curve from time 0 extrapolated to infinity (AUC_0−inf_) and 0–24 h (AUC_0–24_) for motesanib or 0–2 h (AUC_0–2_) for gemcitabine.

### Tumour response

Computed tomography (CT) or magnetic resonance imaging (MRI) for tumour assessments were performed at baseline and at 12-week intervals, or as clinically indicated. In patients with baseline measurable disease tumour response per modified RECIST (Therasse *et al*, 2000) was assessed by the investigator. Complete and partial responses were confirmed using CT or MRI scans no less than 28 days after the initial response.

### Statistical analysis

The safety analysis set included all patients who received ⩾1 dose of motesanib. The pharmacokinetic analysis set included all patients from the safety analysis set who had evaluable plasma samples. The comparative pharmacokinetic analysis subset included patients with evaluable *C*_max_ values following single-dose administration and multiple-dose administration in combination with gemcitabine.

## Results

### Patients

Twenty-six patients were enroled; all received at least one dose of motesanib and one gemcitabine infusion. The demographic and baseline characteristics are summarised in Table 1. The most common tumour types were soft tissue sarcoma (15%), ovarian (12%), pancreatic (12%), and neuroendocrine (8%). Most patients (92%) had advanced disease, and had received prior chemotherapy (65%) or radiation therapy (54%). All 26 patients discontinued treatment with motesanib because of the following reasons: disease progression (*n*=14), withdrawal of consent (*n*=5), administrative decision (*n*=3), adverse event (*n*=2), death (*n*=1), and patient request (*n*=1). Patients received treatment with motesanib on a median of 75 days (minimum–maximum, 14–188), and received a median of 7.5 gemcitabine infusions (minimum–maximum, 2–22).

### Dose escalation, dose-limiting toxicities, and target dose

The first cohort (motesanib 50 mg QD starting dose) enroled 11 patients to have six evaluable patients for the safety review (five patients were deemed unevaluable per protocol criteria). No DLTs occurred. The median treatment duration was 83 days (minimum–maximum, 55–188). Six patients were then enroled in the 125-mg QD cohort. This dose had been previously established as the motesanib maximum-tolerated dose in the first-in-human study (Rosen *et al*, 2007). One DLT of grade 4 neutropenia occurred that was considered (per investigator) to be related to both motesanib and gemcitabine treatment. The 125-mg QD dose was established as the target once-daily dose per protocol definition (see Materials and Methods). A maximum-tolerated dose was not determined in this study. As the 125-mg QD dose was reasonably well tolerated, doses between 50 mg and 125 mg QD were not tested. Patients in the 125-mg QD cohort received motesanib treatment on a median of 53 days (minimum–maximum, 20–181). The 75-mg BID cohort enroled nine patients. No DLTs occurred. One patient had jugular vein thrombosis that was considered by the study site to be not related to motesanib. However, the cohort was discontinued early because an increased incidence of cholecystitis, compared with the general population of cancer patients, was observed at this dose level in other motesanib studies (Amgen Inc., data on file). The median treatment duration in this cohort was 34 days (minimum–maximum, 14–112).

### Adverse events

Sixteen patients (62%) experienced motesanib treatment-related adverse events, including several events of special interest (Table 2). Hypertension was generally manageable with antihypertensive treatment, although two patients (one in the 50-mg QD cohort and one in the 75-mg BID cohort) had motesanib dose reductions to control hypertension, and treatment was withheld in one patient in the 125-mg QD cohort. Two grade 3 motesanib-related adverse events (lethargy, considered related to both motesanib and gemcitabine treatment, and deep vein thrombosis; 75-mg BID cohort) and 1 grade 4-related event (neutropenia; 125-mg QD cohort) occurred. Serious related events were grade 2 dehydration and lethargy (50-mg QD cohort); and grade 3 deep vein thrombosis (75-mg BID cohort). No cases of cholecystitis occurred. Three patients died during the study. The deaths were attributed to disease progression (50-mg QD cohort), renal failure (50-mg QD cohort), and *Pneumocystis jiroveci* pneumonia (75-mg BID cohort), respectively. All deaths were unrelated to motesanib treatment.

### Pharmacokinetics of motesanib

**Figure 1:**
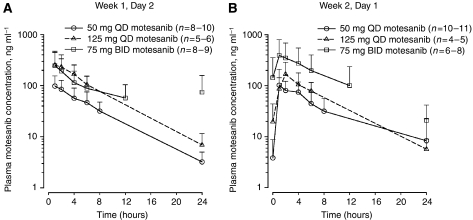
Mean plasma concentration *vs* time profiles for motesanib after single-dose administration (week 1, day 2) (**A**) and multiple-dose administration in combination with gemcitabine (week 2, day 1) (**B**). Data are means (s.d.). BID=two times daily; QD=once daily.

Mean (s.d.) plasma motesanib concentration *vs* time profiles and a summary of pharmacokinetic parameters for intensive pharmacokinetic sampling on day 2 of week 1 and day 1 week 2 are shown in Figure 1 and Table 3, respectively. After single-dose oral administration, motesanib was rapidly absorbed, with an overall median *t*_max_ ranging from 0.5 to 1.5 h for all three dose cohorts. Similar overall median *t*_max_ values (0.83–2.0 h) were observed after multiple-dose administration in combination with gemcitabine. The mean *t*_1/2,z_ values ranged from 3.8 to 5.7 h after single-dose administration and from 4.3 to 5.0 h after multiple-dose administration in combination with gemcitabine; these values did not appear to be dose- or time-dependent.

The mean daily trough plasma concentrations (C_24_) for the 50-mg QD cohort were below the *in vitro* IC_50_ of human umbilical vein endothelial cell (HUVEC) proliferation (IC_50_=4 ng/ml) (Polverino *et al*, 2006) during cycle 1 but were above the IC_50_ during cycle 2. Mean C_24_ values for both week 1 and week 2 were above the IC_50_ for the 125-mg QD cohort, and were above the *in vitro* 90% inhibitory concentration (IC_90_) of HUVEC proliferation (IC_90_=28 ng/ml; Amgen Inc., data on file) for the 75-mg BID cohort.

The *C*_max_ values (week 1) for the 125-mg QD cohort were comparable to those observed in the monotherapy study (Rosen *et al*, 2007). However, motesanib *C*_max_ (except 125 mg QD, week 1) and AUC values for week 1 and 2 in the QD cohorts were slightly lower (<50% decrease) than the values observed in the motesanib monotherapy study.

### Pharmacokinetics of gemcitabine

**Figure 2:**
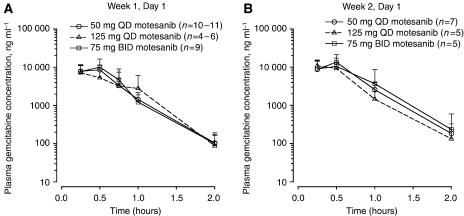
Mean plasma concentration *vs* time profiles for gemcitabine (1000 mg m^−2^ i.v.) administered alone (week 1, day 1) (**A**) or in combination with motesanib (week 2, day 1) (**B**). Data are means (s.d.). BID=two times daily; i.v.=intravenous; QD=once daily.

Mean (s.d.) plasma gemcitabine concentration *vs* time profiles and a summary of pharmacokinetic parameters for intensive pharmacokinetic sampling on day 1 of week 1 and week 2 from all evaluable patients are shown in Figure 2 and Table 4, respectively. Across motesanib dose groups, values for *C*_max_ and AUC_0–2_ in week 1 ranged from 9720 to 10 900 ng ml^−1^ and from 4.72 to 6.27 *μ*g h ml^−1^, respectively; in week 2, these values ranged from 11 400 to 13 800 ng ml^−1^ and from 6.56 to 7.92 *μ*g h ml^−1^, respectively. For all motesanib dose groups, the mean gemcitabine *C*_max_ and AUC values in the same patients were slightly higher (<2-fold) in week 2 compared with week 1.

### Tumour response

Of the 26 patients enroled, one patient in the 125-mg QD dose cohort with stage IV bladder cancer had a confirmed partial response (Table 5). Of patients with a best response of stable disease at 52 days (7 of 26), two had primary diagnoses of pancreatic cancer (50-mg QD cohort); the others had diagnoses of breast cancer (50 mg QD), ovarian cancer (50 mg QD), soft tissue sarcoma (125 mg QD), neuroendocrine cancer (125 mg QD), and other (75 mg BID), respectively. No patients achieved durable stable disease ⩾24 weeks. Forty-six percent of patients (12 of 26) had progressive disease as their best response.

## Discussion

Inhibition of angiogenesis has emerged as an effective therapy with acceptable tolerability in a variety of different solid tumours, including colorectal cancer (Hurwitz *et al*, 2004), NSCLC (Sandler *et al*, 2006), and breast cancer (Miller *et al*, 2007). Recent studies have suggested that treatment with an antiangiogenic agent in combination with cytotoxic chemotherapy can result in increased antitumour efficacy, compared with chemotherapy alone, while maintaining a tolerable safety profile (Miller *et al*, 2005; Giantonio *et al*, 2007; Herbst *et al*, 2007). Motesanib is a highly specific inhibitor of VEGFR1, 2, and 3; of PDGFR, and Kit (Polverino *et al*, 2006), all of which have been implicated as key regulators of angiogenesis, lymphangiogenesis, and tumour cell proliferation (Heinrich *et al*, 2002; Ferrara *et al*, 2003; Yu *et al*, 2003). In the first-in-human monotherapy study, motesanib showed encouraging efficacy and acceptable toxicity in patients with advanced solid tumours (Rosen *et al*, 2007). In the study reported here, combination therapy with motesanib 125 mg QD plus gemcitabine (1000 mg m^−2^) was well tolerated and 125 mg QD was established as the maximum target dose. Adverse events considered related to motesanib were generally of mild to moderate severity. The overall safety profile was similar to that observed in a previous monotherapy study (Rosen *et al*, 2007), and was comparable to results reported from monotherapy studies of other VEGF inhibitors (Yang *et al*, 2003; Herbst *et al*, 2005; Motzer *et al*, 2006) and those from chemotherapy combination studies (Kindler *et al*, 2005; Richly *et al*, 2006; Heymach *et al*, 2007). Similarly, specific adverse events typically associated with VEGF inhibition, such as hypertension, bleeding, or thromboembolic events, did not occur more frequently in the study described here than in monotherapy studies of other VEGF inhibitors (Herbst *et al*, 2005) or in gemcitabine combination studies, such as a phase 2 trial of bevacizumab plus gemcitabine in advanced pancreatic cancer (Kindler *et al*, 2005). Of note, other toxicities, for example, dermatologic toxicities, frequently reported with certain VEGFR and PDGFR inhibitors both as single-agent treatment (Escudier *et al*, 2007) or in combination with cytotoxic chemotherapy (Richly *et al*, 2006) did not occur in this study. Gemcitabine coadministration did not affect the tolerability profile of motesanib in this clinical trial. This finding is consistent with prior studies, which demonstrated that the addition of cytotoxic chemotherapy did not significantly alter the safety profile of other tyrosine kinase inhibitors including sorafenib (Richly *et al*, 2006) and vandetanib (Heymach *et al*, 2007). Although no cases of cholecystitis occurred in this study, the 75-mg BID dose cohort was discontinued early because of an increased risk of cholecystitis in patients who received this dose in other motesanib studies. The cause and relation to motesanib is unknown and is currently under investigation. Acute cholecystitis related to treatment with multikinase inhibitors was earlier reported only once in a patient receiving sunitinib (Motzer *et al*, 2006).

The pharmacokinetics of motesanib and gemcitabine were not markedly affected by coadministration of the other compound. The *C*_max_ and AUC values for motesanib on week 2 compared with week 1 varied across dose groups. Moreover, there was no apparent trend of increasing or decreasing motesanib exposure during coadministration with gemcitabine with increasing doses of motesanib. An effect was not expected because, unlike motesanib, gemcitabine is not metabolised by CYP3A4 (Shipley *et al*, 1992; Mini *et al*, 2006). Exposure to motesanib in patients in the 50-mg QD and 125-mg QD dose cohorts was similar (although slightly lower) to that observed in a previous monotherapy study (Rosen *et al*, 2007). The 75-mg BID dose has not been tested in monotherapy studies and, therefore, no comparison of pharmacokinetic parameters and trough concentrations can be made. Although mean gemcitabine concentrations on week 2 were slightly higher than those on week 1, exposure to gemcitabine, both alone and in combination with motesanib, was no greater than the exposure observed in previous studies investigating gemcitabine monotherapy (Cattel *et al*, 2006; Gietema *et al*, 2006).

Of the 26 patients enroled in this study, one achieved a confirmed partial response and seven achieved stable disease as their best tumour response. Although interpretation of the efficacy results is limited by the small sample size and the exploratory nature of this end point, the data support further exploration in larger studies, which is also encouraged by the recent experience with other combination treatments. Specifically, the addition of VEGF inhibitors to cytotoxic chemotherapy has been shown to enhance antitumour activity, compared with single-agent chemotherapy treatment (Miller *et al*, 2005, 2007; Giantonio *et al*, 2007; Herbst *et al*, 2007; Heymach *et al*, 2007). In particular, preliminary results from a phase 3 study in patients with advanced NSCLC have suggested that improved efficacy can be achieved when combining a VEGF inhibitor with a chemotherapy regimen that includes gemcitabine and cisplatin (Manegold *et al*, 2005). Progression-free survival was longer among patients who received bevacizumab plus gemcitabine/oxaliplatin compared with patients treated with gemcitabine/oxaliplatin plus placebo (6.7 *vs* 6.1 months) (Manegold *et al*, 2005). On the other hand, preliminary results from another phase 3 study suggest that treatment of advanced pancreatic cancer with bevacizumab and gemcitabine does not improve survival, although the proportion of patients with an objective response or stable disease was slightly greater (54 *vs* 47%) (Kindler *et al*, 2007). Interestingly, preclinical evidence has suggested that inhibition of PDGFR, which regulates vascular survival and is expressed in most common solid tumours, may play an important role in enhancing the therapeutic effect of cytotoxic chemotherapy (Pietras *et al*, 2002). The potential efficacy of motesanib, which inhibits both VEGFR and PDGFR, in combination with gemcitabine or chemotherapy in general, warrants further investigation in larger studies.

In conclusion, treatment with motesanib 125 mg QD plus gemcitabine was well tolerated, with adverse event and pharmacokinetic profiles similar to those observed in motesanib monotherapy studies. The 125-mg QD dose in combination with cytotoxic chemotherapy is being investigated in larger studies to further evaluate safety and efficacy.

## Figures and Tables

**Table 1:** Demographic and clinical characteristics of study patients

	**Motesanib dose cohort**			
**Characteristics**	**50 mg QD (*n*=11)**	**125 mg QD (*n*=6)**	**75 mg BID (*n*=9)**	**All patients (*n*=26)**
Men, n (%)	5 (45)	3 (50)	6 (67)	14 (54)
				
*Race*, n (%)				
White	11 (100)	6 (100)	8 (89)	25 (96)
Hispanic or Latino	0 (0)	0 (0)	1 (11)	1 (4)
Median age, years (minimum–maximum)	57.0 (34–76)	49.5 (25–66)	57.0 (32–77)	56.5 (25–77)
				
*ECOG performance status*, n (%)				
0	5 (45)	3 (50)	4 (44)	12 (46)
1	5 (45)	3 (50)	5 (56)	13 (50)
2	1 (9)	0 (0)	0 (0)	1 (4)
				
*Tumour type*, n (%)				
Soft-tissue sarcoma	2 (18)	1 (17)	1 (11)	4 (15)
Ovarian	2 (18)	1 (17)	0 (0)	3 (12)
Pancreatic	2 (18)	0 (0)	1 (11)	3 (12)
Neuroendocrine	1 (9)	1 (17)	0 (0)	2 (8)
Bile duct	0 (0)	0 (0)	1 (11)	1 (4)
Bladder	0 (0)	1 (17)	0 (0)	1 (4)
Breast	1 (9)	0 (0)	0 (0)	1 (4)
Colon	0 (0)	1 (17)	0 (0)	1 (4)
Oesophageal	0 (0)	0 (0)	1 (11)	1 (4)
Gall bladder	0 (0)	0 (0)	1 (11)	1 (4)
Non-small cell lung	0 (0)	0 (0)	1 (11)	1 (4)
Other^a^	3 (27)	1 (17)	3 (33)	7 (27)
				
*Prior chemotherapy*, n (%)				
0	2 (18)	3 (50)	4 (44)	9 (35)
1	1 (9)	1 (17)	2 (22)	4 (15)
2	5 (45)	0 (0)	2 (22)	7 (27)
⩾3	3 (27)	2 (33)	1 (11)	6 (23)
				
*Prior radiation therapy*, n (%)				
0	6 (55)	2 (33)	4 (44)	12 (46)
1	2 (18)	4 (67)	4 (44)	10 (38)
2	1 (9)	0 (0)	1 (11)	2 (8)
3	2 (18)	0 (0)	0 (0)	2 (8)

BID=two times daily; ECOG=Eastern Cooperative Oncology Group; QD=once daily.

a One patient in the 125-mg QD dose cohort had Burkitt's lymphoma.

**Table 2:** Motesanib treatment-related adverse events

	**Motesanib dose cohort**			
	**50 mg QD (*n*=11)**	**125 mg QD (*n*=6)**	**75 mg BID (*n*=9)**	**All patients (*n*=26)**
*Patients with motesanib-related adverse events*, n (%)	6 (55)	4 (67)	6 (67)	16 (62)
Lethargy	3 (27)	0 (0)	3 (33)	6 (23)
Diarrhoea	2 (18)	1 (17)	1 (11)	4 (15)
Fatigue	0 (0)	1 (17)	2 (22)	3 (12)
Headache	1 (9)	2 (33)	0 (0)	3 (12)
Nausea	1 (9)	1 (17)	1 (11)	3 (12)
Anorexia	0 (0)	0 (0)	2 (22)	2 (8)
Vomiting	1 (9)	0 (0)	1 (11)	2 (8)
				
*Patients with motesanib-related adverse events of interest*, n (%)^a^				
Hematemesis	0 (0)	0 (0)	1 (11)	1 (4)
Grade ⩾3	0 (0)	0 (0)	0 (0)	0 (0)
Deep vein thrombosis	0 (0)	0 (0)	1 (11)	1 (4)
Grade 3	0 (0)	0 (0)	1 (11)	1 (4)
Hypertension	0 (0)	0 (0)	1 (11)	1 (4)
Grade ⩾3	0 (0)	0 (0)	0 (0)	0 (0)
Neutropenia	0 (0)	1 (17)	0 (0)	1 (4)
Grade 4	0 (0)	1 (17)	0 (0)	1 (4)

BID=two times daily; QD=once daily.

a Including highest worst grade.

**Table 3:** Pharmacokinetic parameter estimates of motesanib after single-dose administration and after multiple-dose with gemcitabine administration

**Parameter**	**After single-dose motesanib administration (week 1, day 2)**		**After multiple-dose motesanib administration in combination with gemcitabine (week 2, day 1)**	
*50 mg QD*	*n*		*n*	
*t*_max_, h (range)	10	0.5 (0.25–6.0)	11	1.0 (0.28–4.0)
*C*_max_, ng ml^−1^ (s.d.)	10	208 (147)	11	176 (136)
C_24_, ng ml^−1^ (s.d.)	8	3.21 (1.80)	10	8.36 (14.1)
AUC_0–24_, *μ*g h ml^−1^ (s.d.)	8	0.630 (0.208)	10	0.764 (0.526)
AUC_0–inf_, *μ*g h ml^−1^ (s.d.)	8	0.656 (0.219)	NR	NR
*t*_1/2,z_, h (s.d.)	8	5.17 (0.873)	7	4.32 (0.668)
CL/F, L/h (s.d.)	8	83.7 (26.0)	10	106 (83.4)
				
*75 mg BID* a	*n*		*n*	
*t*_max_, h (range)	9	0.57 (0.25–2.0)	8	0.83 (0.25–4.0)
*C*_max_, ng ml^−1^ (s.d.)	9	345 (266)	8	515 (463)
C_12_, ng ml^−1^ (s.d.)	9	57.2 (46.5)	7	100 (137)
C_24_, ng ml^−1^ (s.d.)	8	74.7 (83.7)	6	29.5 (20.9)
AUC_0–24_, *μ*gh ml^−1^ (s.d.)	9	2.76 (1.95)	7	4.96 (5.83)
AUC_0–inf_, *μ*gh ml^−1^ (s.d.)	NR	NR	NR	NR
*t*_1/2,z_, h (s.d.)	7	5.65 (2.21)	7	4.99 (1.98)
CL/F, L/h (s.d.)	9	152 (235)	7	239 (468)
				
*125 mg QD*	*n*		*n*	
*t*_max_, h (range)	6	1.5 (0.33–4.0)	5	2.0 (0–2.0)
*C*_max_, ng ml^−1^ (s.d.)	6	421 (194)	5	184 (96.1)
C_24_, ng ml^−1^ (s.d.)	5	6.90 (4.60)	4	5.71 (3.38)
AUC_0–24_, *μ*g h ml^−1^ (s.d.)	5	1.90 (0.450)	4	1.24 (0.535)
AUC_0–inf_, *μ*g h ml^−1^ (s.d.)	6	1.70 (0.734)	NR	NR
*t*_1/2,z_, h (s.d.)	6	3.80 (1.40)	4	4.60 (0.594)
CL/F, L/h (s.d.)	6	102 (88.1)	4	124 (74.4)

AUC_0–inf_=area under the plasma concentration versus time curve from time 0 to infinity; AUC_0–24_=area under the plasma concentration versus time curve from time 0–24 h postdose; BID=two times daily; CL/F=apparent clearance; *C*_max_=maximum observed concentration after dosing; C_12_=observed concentration at 12 h postdose; C_24_=observed concentration at 24 h postdose; NR=not reported; QD=once daily; *t*_max_=time of *C*_max_; *t*_1/2,z_=estimated terminal elimination half-life.

a For the 75-mg BID dose cohort, *t*_1/2,z_ values were estimated based on the terminal slope after the first dose on week 1 or 2, AUC_0–24_ values were estimated using 2 × AUC_0–12_, and CL/F was estimated by total daily dose/(2 × AUC_0–12_).

**Table 4:** Pharmacokinetic parameter estimates for 1000 mg m^−2^ intravenous gemcitabine administered alone (week 1, day 1) or in combination with motesanib (week 2, day 1)

**Parameter**	**Administered alone (week 1, day 1)**		**Administered in combination with motesanib (week 2, day 1)**	
50 mg QD	*n*		*n*	
*t*_max_, h (range)	11	0.5 (0.13–0.75)	7	0.5 (0.25–0.50)
*C*_max_, ng ml^−1^ (s.d.)	11	9720 (3240)	7	13 800 (7530)
C_2_, ng ml^−1^ (s.d.)	11	104 (60)	7	183 (143)
AUC_0–2_, *μ*g h ml^−1^ (s.d.)	11	5.21 (1.57)	7	7.92 (3.59)
75 mg BID	*n*		*n*	
*t*_max_, h (range)	9	0.5 (0.25–0.75)	5	0.5 (0.5–1.0)
*C*_max_, ng ml^−1^ (s.d.)	9	10 900 (6230)	5	12 800 (4080)
C_2_, ng ml^−1^ (s.d.)	9	101 (92)	5	234 (360)
AUC_0–2_, *μ*g h ml^−1^ (s.d.)	9	6.27 (2.94)	5	7.09 (2.37)
125 mg QD	*n*		*n*	
*t*_max_, h (range)	6	0.38 (0.18–0.75)	5	0.25 (0.25–0.50)
*C*_max_, ng ml^−1^ (s.d.)	6	10 000 (4460)	5	11 400 (4010)
C_2_, ng ml^−1^ (s.d.)	6	88 (88)	5	133 (96)
AUC_0–2_, *μ*g h ml^−1^ (s.d.)	6	4.72 (2.18)	5	6.56 (2.33)

AUC_0–2_=area under the plasma concentration versus time curve from time 0–2 h postdose; BID=two times daily; C_2_=observed concentration at 2 h postdose; *C*_max_=maximum observed concentration after dosing; QD=once daily; *t*_max_=time of *C*_max_.

**Table 5:** Best tumour response per modified RECIST as assessed by investigator

	**Motesanib dose cohort**			
	**50 mg QD (*n*=11)**	**125 mg QD (*n*=6)**	**75 mg BID (*n*=9)**	**All patients (*n*=26)**
Patients with measurable disease at baseline, *n* (%)	11 (100)	6 (100)	9 (100)	26 (100)
				
*Response assessment,* n (%)				
Confirmed complete response^a^	0 (0)	0 (0)	0 (0)	0 (0)
Confirmed partial response^a^	0 (0)	1 (17)	0 (0)	1 (4)
Stable disease	4 (36)	2 (33)	1 (11)	7 (27)
Progressive disease	7 (64)	3 (50)	2 (22)	12 (46)
Not done	0 (0)	0 (0)	6 (67)	6 (23)
Confirmed objective response^b^, % (95% CI)	0 (0.0–28.5)	17 (0.4–64.1)	0 (0.0–33.6)	4 (0.1–19.6)

a Patients with a response assessment of complete response or partial response that was not confirmed at least 4 weeks later are reported as stable disease.

b Objective response is defined as a tumour response assessment of either confirmed complete response or confirmed partial response.
